# An Electrochemical Sensor Based on a Nitrogen-Doped Carbon Material and PEI Composites for Sensitive Detection of 4-Nitrophenol

**DOI:** 10.3390/nano12010086

**Published:** 2021-12-29

**Authors:** Xue Nie, Peihong Deng, Haiyan Wang, Yougen Tang

**Affiliations:** 1 Key Laboratory of Functional Metal-Organic Compounds of Hunan Province, Key Laboratory of Functional Organometallic Materials of Hunan Provincial Universities, College of Chemistry and Material Science, Hengyang Normal University, Hengyang 421008, China; hynuniexue@126.com; 2 College of Chemistry and Chemical Engineering, Central South University, Changsha 410083, China; wanghy419@csu.edu.cn

**Keywords:** nitrogen-doped carbon material, polyethyleneimine, 4-nitrophenol, voltammetric detection, water sample

## Abstract

A glassy carbon electrode (GCE) was modified with nitrogen-doped carbon materials (NC) and polyethyleneimine (PEI) composites to design an electrochemical sensor for detecting 4-nitrophenol (4-NP). The NC materials were prepared by a simple and economical method through the condensation and carbonization of formamide. The NC materials were dispersed in a polyethyleneimine (PEI) solution easily. Due to the excellent properties of NC and PEI as well as their synergistic effect, the electrochemical reduction of the 4-NP on the surface of the NC–PEI composite modified electrode was effectively enhanced. Under the optimized conditions, at 0.06–10 μM and 10–100 μM concentration ranges, the NC–PEI/GCE sensor shows a linear response to 4-NP, and the detection limit is 0.01 μM (the signal-to-noise ratio is three). The reliability of the sensor for the detection of 4-NP in environmental water samples was successfully evaluated. In addition, the sensor has many advantages, including simple preparation, fast response, high sensitivity and good repeatability. It may be helpful for potential applications in detecting other targets.

## 1. Introduction

With the development of industrialization, the harmful effects of phenolic compounds on the environment are more and more serious. Phenolic compounds are the products of many industrial processes, which are often released into the environment, and exist in soil and wastewater at the same time. Among all kinds of phenolic compounds, 4-nitrophenol (4-NP) is one of the priority organic pollutants listed by the U.S. Environmental Protection Agency [1]. The detection of 4-NP is an important topic in environmental pollution control. An important intermediate chemical, 4-NP is used in the production of pesticides, such as parathion, fenitrothion and methyl parathion [2]. However, 4-NP is highly toxic to mammals, fish and other aquatic organisms. It has been reported to be carcinogenic, mutagenic and cytoembryotoxic [3]. Therefore, the monitoring of 4-NP in the environment has been of wide concern, and a variety of analytical methods have been reported. At present, the analytical techniques used, such as chemiluminescence [4], fluorescence [5], high-performance liquid chromatography [6], capillary zone electrophoresis [7], gas chromatography–mass spectrometry [8], etc., often require complex sample pretreatment, well-trained technicians, expensive instruments and poisonous solvents.

In recent years, due to its advantages of eco-friendliness, high sensitivity, good selectivity and low cost, an electrochemical method has been widely used in the determination of pollutants, small biomolecules, food additives and heavy metal ions [9,10,11,12,13,14,15,16,17,18,19]. A molecule with high electrochemical activity, 4-NP can be easily reduced in the process of an electrochemical reaction. Therefore, various electrochemical sensors for 4-NP detection have been proposed [3,20,21,22,23,24,25,26,27] Although there are many kinds of electrochemical sensors for determining 4-NP, most of the electrochemical sensors for 4-NP detection are limited due to the complex electrode preparation, long response time, in-sufficient selectivity and poor stability. Therefore, it is still a challenge to prepare novel electrochemical sensors with excellent electrocatalytic properties by simple methods.

Carbon materials are widely used in electrochemical sensors because of their good conductivity, stable chemical properties and wide potential window. Nowadays, a variety of carbon materials with different structures have been successfully prepared, such as fullerene, carbon nanotubes, graphene, carbon nanofilms, carbon nanoparticles and porous carbon materials. Different structures of carbon materials lead to different surface properties, which makes them have a wide range of applications in materials, energy, electronic, biomedical and other fields. However, carbon has poor hydrophilicity and low surface activity, which seriously limits its functionalization and application. In recent years, the use of nitrogen-doped carbon materials to obtain unique properties has become a research hotspot [28,29,30,31]. When nitrogen is doped into carbon materials, it can not only enhance the conductivity of carbon materials, but also enhance the hydrophilicity and increase the number of surface active sites of carbon materials, which makes the nitrogen-doped carbon materials have more excellent physical and chemical properties [32]. At present, nitrogen-doped carbon materials are mainly prepared by an in situ doping method and post-treatment method. In the process of in situ doping, a special precursor containing a nitrogen atom is introduced into the synthesis of the carbon materials. After carbonization, the nitrogen atoms enter into the carbon materials. Commonly used nitrogen-containing precursors include polyimide [33], ethylenediamine [34], ionic liquid [35], melamine [36], polypyrrole [37], acetonitrile [38], urea [39] and nitrogen-containing metal organic framework compounds (MOFs) [40]. The post-treatment is to use some nitrogen-rich materials as nitrogen sources to modify the pre-synthesized carbon materials [41,42,43]. However, most of these nitrogen-doped carbon materials have a low nitrogen content and complicated synthesis steps.

In this study, a novel nitrogen-doped carbon material (NC) was prepared by using formamide as the nitrogen and carbon sources. Polyethyleneimine (PEI) is a kind of water-soluble polymer rich in amino groups, which is a good dispersant for the NC material into an aqueous solution. Therefore, this PEI–NC composite can be uniformly coated on the surface of a glassy carbon electrode. In addition, the composite film can be firmly attached to the electrode surface due to the excellent viscosity of the PEI, which greatly improves the repeatability and stability of the sensor. The electrochemical behavior of 4-NP on the PEI–NC/GCE was studied in detail. This PEI–NC/GCE sensor has been successfully applied for determining 4-NP in environmental water samples. Based on this, a simple and sensitive method for determining 4-NP is proposed.

## 2. Experimental

### 2.1. Chemicals and Reagents

Formamide (FA) and 4-nitrophenol (4-NP) were purchased from Sinopharm Chemical Reagent Co., Ltd., Shanghai, China. Polyethyleneimine (PEI) (25000) was obtained from Sigma-Aldrich (St. Louis, MO, USA). The 4-NP stock solution (1.0 mM) was prepared by dissolving 4-NP into ethanol. Working solutions were freshly prepared by appropriate dilution of the stock solution. During the whole electrochemical measurements, 0.2 M H_2_SO_4_ was used as the supporting electrolyte. All reagents were of analytical grade and used directly. All solutions were prepared with ultrapure water with resistivity >18 MΩ cm.

### 2.2. Apparatus

The surface morphologies of the NC material and NC–PEI composite were characterized by scanning electron microscope (Zeiss Sigma 300, Gemini, Jena, Germany) and transmission electron microscope (JEOL JEM-2100, Tokyo, Japan). X-ray diffraction (XRD) patterns were collected with a powder X-ray diffractometer (D8 Advance, Bruker AXS, Karlsruhe, Germany) with Cu Kα radiation (λ = 0.154056 nm). X-ray photoelectron spectroscopy (Thermo Electron ESCALAB250 XPS Spectrometer) was used to characterize the NC nanohybrid composition and element state. C1s (284.8 eV) was utilized for calibrating the binding energies of the acquired XPS spectra. Cyclic voltammetry (CV) were evaluated with an electrochemical workstation (CHI 660E, Shanghai Chenhua Instrument Co. Ltd., Shanghai, China) with a scan rate of 0.1 mV s ^−1^ in the potential range of −0.6 to 0.4 V. Second-order derivative linear sweep voltammetry (SDLSV) was collected with a model JP-303E polarographic analyzer (Chengdu Instrument Factory, Chengdu, China) for quantitative analysis. A conventional three-electrode system was used throughout electrochemical measurements, which consists of an unmodified or modified GCE (id = 3.0 mm) as the working electrode, a platinum wire as the counter electrode and a saturated calomel electrode (SCE) as the reference electrode. For pH measurements, the pH-3c Model pH meter (Shanghai Leichi Instrument Factory, Shanghai, China) with a combined glass electrode was used.

### 2.3. Synthesis of NC Material

Because of the amino and carbonyl groups in formamide molecules, the Schiff’s base reaction between the amino groups and carbonyl groups of the formamide molecules took place by solvothermal method to form a one-dimensional C=N molecular chain. The preparation of NC is presented in the following steps: formamide (30 mL) was added to a 50 mL Teflon-lined autoclave. After solvothermal self-polymerization reaction at 180 °C for 12 h, a brown-black product was taken out by high speed centrifugation; washed with ultrapure water and ethanol three times each; and vacuum dried at 60 °C overnight. The collected product was then placed in a corundum boat with a cover, and the pyrolysis mode of a tubular furnace was set for two-step pyrolysis: In the first step, the temperature was set at 400 °C for 2 h, and the heating rate was 5 °C min^−1^. In the second step, the temperature was set at 900 °C for 2 h, and the heating rate was 10 °C min^−1^. Finally, the brown-black sample carried in a corundum boat was pyrolyzed in a N_2_ atmosphere, and the N-doped carbon material (denoted as NC) was prepared. 

### 2.4. Fabrication of NC–PEI/GCE

**Scheme 1:**
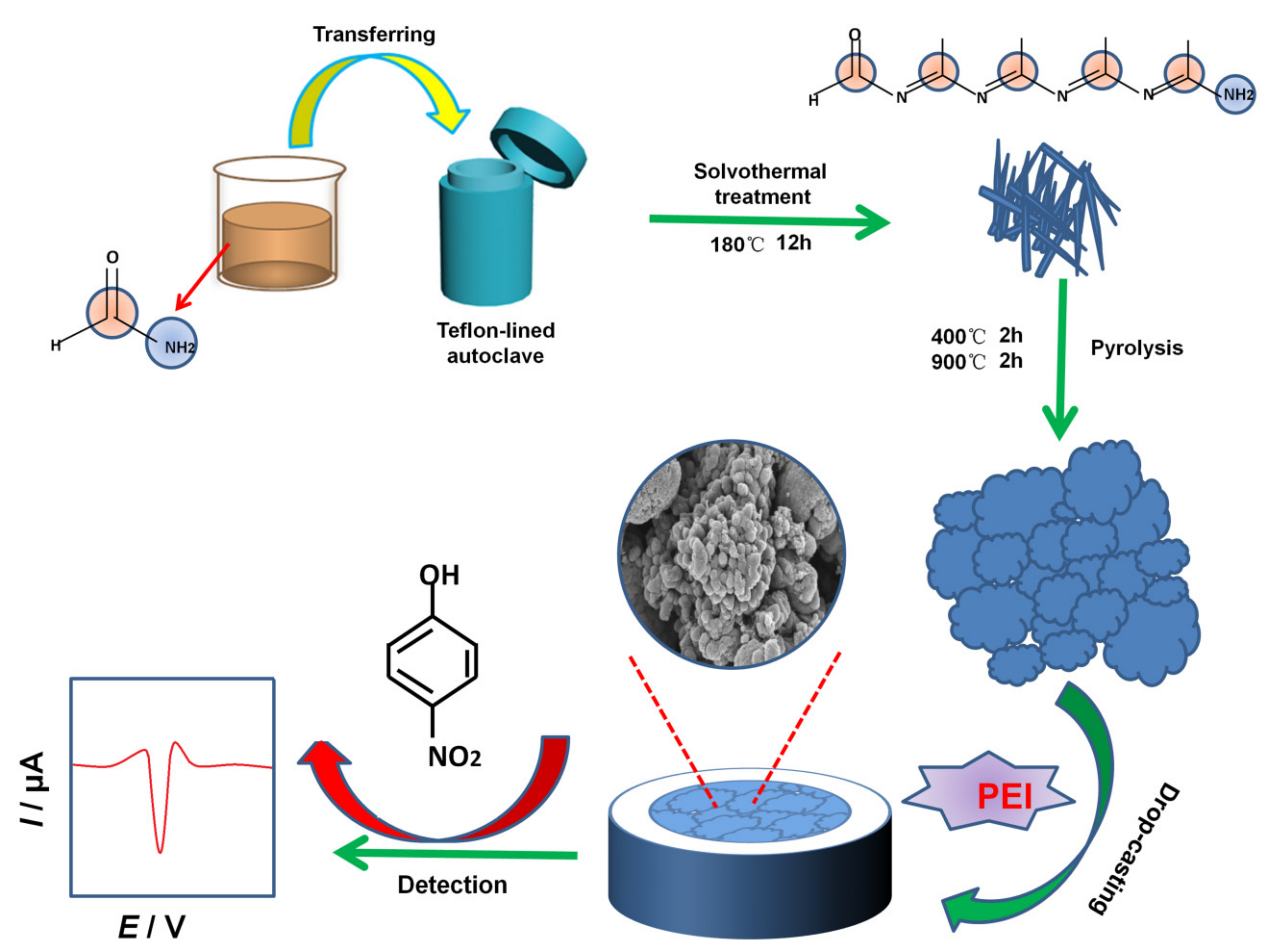
Synthesis of the NC material and the construction of the NC–PEI/GCE-based electrochemical 4-NP sensor.

Before modifying the glassy carbon electrode (GCE, 3 mm in diameter), the exposed GCE was polished into a mirror-like shape with 0.05 μm alumina powder slurry, washed thoroughly with ultrapure water and ethanol for 1 min each in turn, and then dried under an infrared lamp. To form a 1.0 mg mL^−1^ solution, 10.0 mg of PEI were added into 10 mL water. Then, 1.0 mg NC material was added to 1.0 mL PEI aqueous solution (1.0 mg mL^−1^), and the NC material was uniformly dispersed by ultrasonic dispersion for 3 min. Finally, 5.0 μL NC–PEI suspension was dropped onto the polished GCE surface and dried under an infrared lamp to prepare the NC–PEI/GCE, which was directly used as the working electrode for the detection of 4-NP. As a comparison, the pure NC (dispersed in water)-modified electrode (denoted as NC/GCE) and the PEI film-modified GCE (denoted as PEI/GCE) were prepared by similar process. The fabricated modified electrodes were stored in air when not in use. Scheme 1 gives the preparation process of the NC–PEI/GCE.

### 2.5. Procedure 

For voltammetric measurements, firstly, an appropriate volume of 4-NP was added into an electrochemical cell of 10 mL containing 0.2 M H_2_SO_4_ solution. The three-electrode system was then immersed in the solution. The measurement was carried out after an 150-s accumulation at the potential of 0.2 V. Cyclic voltammetry (CV) was measured with a scan rate of 0.1 V s^−1^ in the potential range of −0.6 to 0.4 V, and second-order derivative linear sweep voltammetry (SDLSV) was measured in the potential range of 0.2 V to −0.7 V. After each measurement, the modified electrode was immersed in 0.2 M H_2_SO_4_ solution and scanned several times in the same potential range to obtain a new electrode surface. The same procedure was applied for water sample analysis. All the electrochemical experiments were carried out at room temperature, and oxygen was removed from the test solution by introducing nitrogen before measurement.

## 3. Results and Discussion

### 3.1. Characterization of NC and NC–PEI Composite

**Figure 1:**
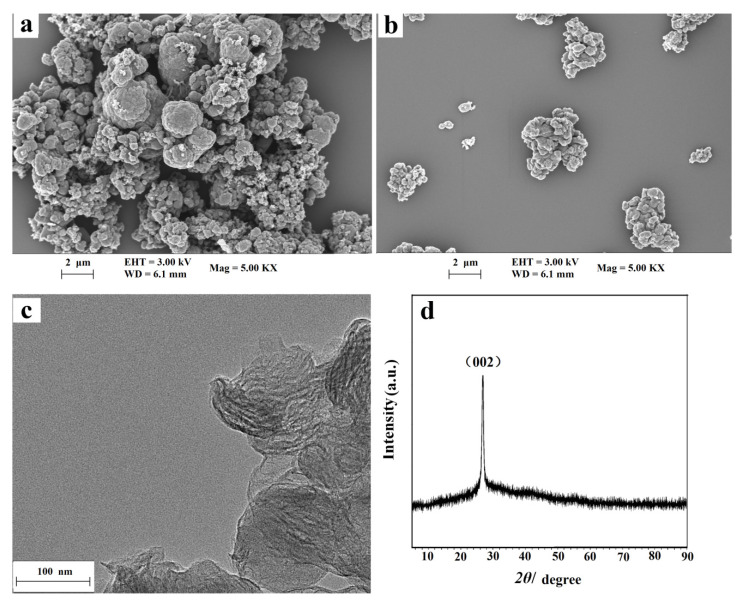
SEM images of NC nanohybrid (**a**) and NC–PEI composite (**b**); TEM image of NC nanohybrid (**c**) and XRD pattern of NC nanohybrid (**d**).

The surface morphologies of the NC and NC–PEI composite were studied by scanning electron microscopy (SEM). As shown in Figure 1a, the NC material was nodular, and its surface is very rough, similar to broccoli. Compared with the NC material, the typical morphology of NC–PEI is almost the same. However, Figure 1b shows that the dispersion of NC in in PEI solution is greatly improved, which indicates that the addition of the PEI has no effect on the morphology of the material, but only improves the dispersion of the NC material in water. From the TEM image of NC in Figure 1c, obvious two-dimensional folds and overlapping nanosheets can be observed. The crystal structure of the synthesized NC material was analyzed by XRD. It can be seen from Figure 1d that the NC material has a peak at 2 θ = 26.6°, which corresponds to the typical peak (002) of the graphite phase of carbon nitride. The XRD result indicated that the NC material has good crystallinity and high purity. 

**Figure 2:**
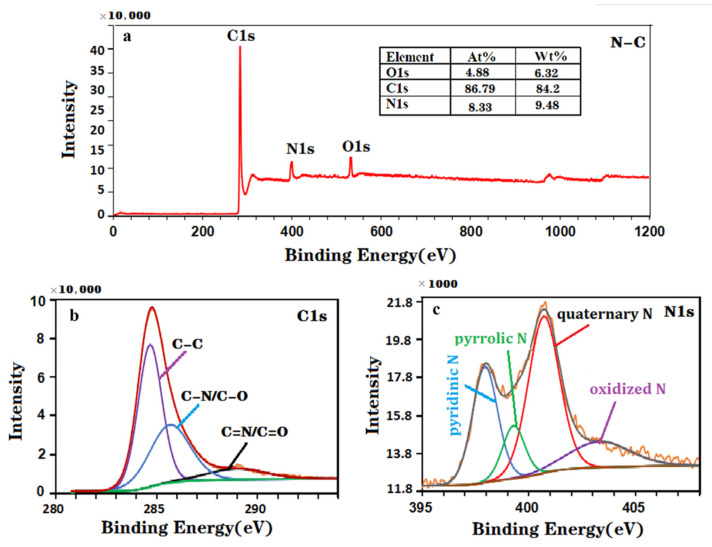
XPS spectra of the NC nanohybrid: (**a**) survey spectrum, (**b**) C1s spectrum and (**c**) N1s spectrum.

The elemental composition and chemical state of the material were also investigated by XPS, as shown in Figure 2a–c. The XPS spectra of the NC nanohybrid (Figure 2a) revealed that the atomic percentages of C, O and N in this sample are 86.79%, 4.88% and 8.33%, respectively. In addition, the three characteristic peaks of C-C (284.6 eV), C-O/C-N (285.8 eV) and N-C=N/C=O (288.9 eV) in the high-resolution C1s spectrum (Figure 2b) confirmed that the N element has been successfully doped into the carbon material [44]. Four characteristic peaks of pyridine N (398.1 eV), pyrrole N (399.4 eV), graphite N (400.8 eV) and oxidation state N (403.4 eV) were found in the high-resolution N 1 s spectra (Figure 2c). The above XPS results show that the synthesized NC nanohybrid contains nitrogen and carbon elements, which further verifies the effectiveness of the XRD results. Generally, the electrochemical performance of nanocomposites can be improved by doping the N element in carbon nanomaterials. Therefore, N-doped carbon nanocomposites can effectively improve the performance of modified electrodes [45,46].

### 3.2. Electrochemical Characterization of Different Electrodes

**Figure 3:**
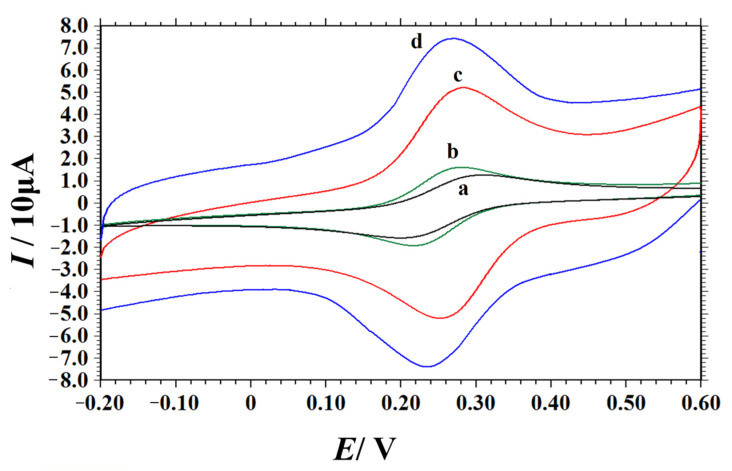
Cyclic voltammograms of 1.0 mM K_3_[Fe(CN)_6_] in 0.5 M KCl solution obtained at bare GCE (a), PEI/GCE (b), NC/GCE (c) and NC–PEI/GCE (d). Scan rate: 0.1 V s^−1^.

The electrochemical behaviors of different electrodes (bare GCE, PEI/GCE, NC/GCE and NC–PEI/GCE) were investigated using potassium ferricyanide as probe by CV technique. The corresponding cyclic voltammograms (Figure 3) showed that the current of oxidation peak (*I*_pa_) and reduction peak (*I*_pc_) of K_3_[Fe(CN)_6_] on bare GCE electrodes were 12.53 μA and 13.95 μA, respectively, and the peak-to-peak separation (Δ*E*_p_) was 110 mV (curve a). Compared with the bare GCE, the redox peak current of K_3_[Fe(CN)_6_] on the PEI/GCE was slightly increased (*I*_pa_ = 16.87 μA, *I*_pc_ = 17.34 μA, curve b). This may be due to the good interaction of the polymeric cation in the PEI with Fe(CN)_6_^3-/4-^ anions. At the NC/GCE, the electrochemical response of K_3_[Fe(CN)_6_] is further enhanced. The peak current is greatly increased (*I*_pa_ = 22.82 μA, *I*_pc_ = 22.63 μA), and the Δ*E*_p_ decreased to 44 mV (curve c). This is mainly because high N content doping in NC materials changes the structure and charge density of the carbon materials and increases the electron density and electron migration rate on the surface of the carbon materials [47,48,49]. At the same time, N-doping provides sufficient active centers for catalytic reaction. Compared with the former three electrodes, the largest redox peak currents of K_3_[Fe(CN)_6_] were obtained by the NC–PEI/GCE (*I*_pa_ = 37.58 μA, *I*_pc_ = 37.27 μA), which may be closely related to the large specific surface area, good electrical conductivity of the NC composites and the good dispersibility and adsorption capacity of PEI, as well as the synergistic effect between them. According to the Randles–Sevcik equation [50], the effective active areas of different electrodes can be calculated:

(1) $$I_{p}=\left(2.69\times 10^{5}\right)n^{3/2}D^{1/2}ACv^{1/2}$$

In the above formula, *I_p_* is the peak current (A), *n* is the number of electrons transfer, *D* is the diffusion coefficient of K_3_[Fe(CN)_6_] (*D* = 6.3 × 10^−6^ cm^2^ s^−1^) [51], *A* is the electrochemical active area (cm^2^), *C* is the concentration of K_3_[Fe(CN)_6_] (mol cm^−3^) and *v* is the scan rate (V s^−1^). Combined with the slope of the above linear equation, the effective active areas of the bare GCE, PEI/GCE, NC/GCE and NC–PEI/GCE were calculated to be 0.065 cm^2^, 0.081 cm^2^, 0.106 cm^2^ and 0.175 cm^2^, respectively. These results clearly showed that the electrochemical active area of the electrode was effectively increased by the NC-–PEI/GCE, which is mainly due to the good dispersion effect of PEI on the NC material. 

### 3.3. Voltammetric Behavior of 4-NP on Different Electrodes

**Figure 4:**
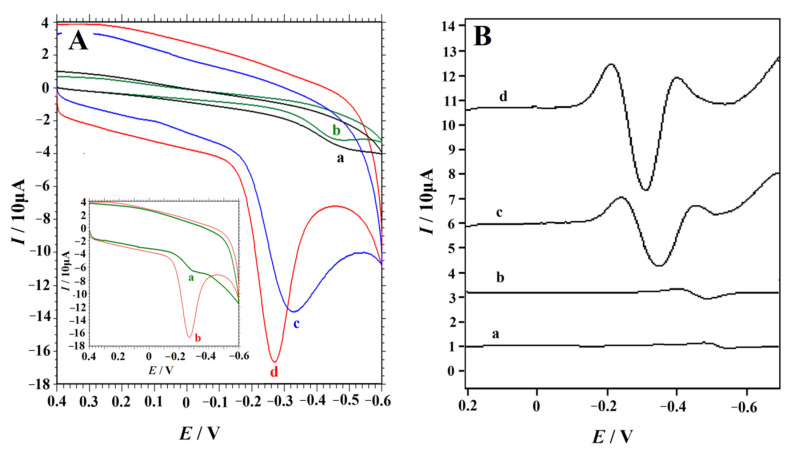
Cyclic voltammograms (**A**) and second-derivative linear sweep voltammograms (**B**) of 0.1 mM 4-NP in 0.2 M H_2_SO_4_ solution obtained at bare GCE (curve a), PEI/GCE (curve b), NC/GCE (curve c) and NC–PEI/GCE (curve d) after accumulating at 0.2 V for 90 s, scan rate: 0.1 V s^−1^. Inset of a: CV curves of 0.1 mM 4-NP in 0.2 M H_2_SO_4_ solution without accumulation (a) and after accumulation for 90 s (b).

Accumulation can improve the amount of 4-NP on the electrode surface, and then obviously improve the determining sensitivity. The cyclic voltammograms of 0.1 mM 4-NP in 0.2 M H_2_SO_4_ solution obtained at the NC–PEI/GCE without accumulation and with accumulation (90 s) were shown in the insert of Figure 4A. It was obvious that after accumulation, the CV responses of 0.1 mM 4-NP increased dramatically. In order to demonstrate the unique and excellent performance of NC–PEI/GCE in the determination of 4-NP, the CV curves of 0.1 mM 4-NP on the bare GCE, PEI/GCE, NC/GCE and NC–PEI/GCE were compared after accumulating at 0.2 V for 90 s. According to the currently accepted mechanism for the electroreduction in aromatic and heteroaromatic nitro compounds [52,53,54], the reduction peak is attributed to the reduction of p-nitrophenol to the corresponding p-(hydroxyamino)phenol. As shown in Figure 4A, on the bare GCE (curve a), an inconspicuous reduction peak appeared at the potential of −0.493 V, indicating a slow electron transfer kinetic. The peak current increased slightly on the PEI/GCE under the same conditions (curve (b)), indicating that the reduction of 4-NP on the PEI/GCE is more favorable, which may be due to the hydrogen bonding interactions between the amino group of PEI and the hydroxyl group of 4-NP. The curves (c) and (d) confirmed the catalytic performance of the NC/GCE and NC–PEI/GCE for 4-NP reduction. Under the same conditions, the reduction peak current of 4-NP on the NC/GCE (85.98 μA) was significantly greater than that on the bare GCE or on PEI/GCE, and the peak potential also shifted positively (−0.327 V) (curve (c)). This may be attributed to the unique characteristics of NC such as a higher charge density on the surface of materials as well as good electrical conductivity because of the large specific surface area and higher N content of NC materials [47,48,49]. When the NC–PEI/GCE was used, a maximum sharp 4-NP reduction peak (117.4 μA) appeared at −0.270 V, which indicates that NC and PEI have synergistic enhancement effect on 4-NP. The highest peak current and the lowest reduction overpotential are the evidence that the NC–PEI composite has good electrocatalytic performance for 4-NP. Due to the excellent properties of NC and PEI, the accumulation efficiency of 4-NP on the electrode surface is greatly improved, and the electron transfer rate on the electrode surface is accelerated, which is beneficial to the sensitive detection of 4-NP on the NC–PEI/GCE.

To further illustrate the performance of different electrodes on 4-NP detection, the SDLSV response of different electrodes to 0.1 mM 4-NP in 0.2 M H_2_SO_4_ solution under the same conditions was collected (Figure 4B). As an electrochemical technique with high signal-to-noise ratio and high sensitivity, SDLSV is widely used in quantitative analysis [55,56,57]. The voltammograms of 0.1 mM 4-NP obtained at the bare GCE, PEI/GCE, NC/GCE and NC–PEI/GCE were compared. The results showed that NC–PEI composites have the best effect on 4-NP reduction. The 4-NP reduction peak current on the NC–PEI/GCE is about two-fold higher than that on the NC/GCE. Meanwhile, it was 22-fold and 13-fold higher than that on the bare GCE and PEI/GCE. These phenomena confirm that NC–PEI composites can improve the electrocatalytic reduction rate of 4-NP, which can be attributed to the combined advantages of the composites, including the good catalytic activity of NC materials with 4-NP and the hydrogen bonding interaction of PEI with the NC materials and 4-NP, which could facilitate the improvement of the electron transfer rate and the accumulation efficiency of 4-NP.

### 3.4. Optimization of the Determination Conditions

**Figure 5:**
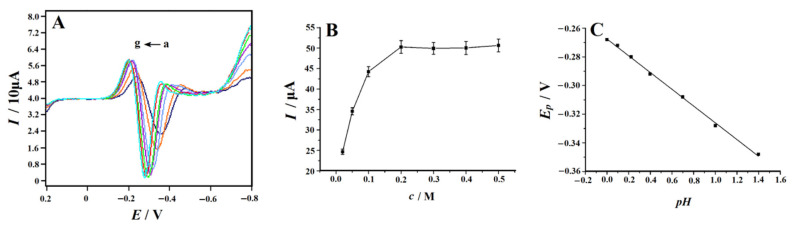
(**A**) Second-derivative linear sweep voltammograms of 0.1 mM 4-NP recorded on the NC–PEI/GCE at a scan rate of 0.1 V s^−1^ in H_2_SO_4_ solution with different concentrations (from a to g are 0.02, 0.05, 0.1, 0.2, 0.3, 0.4 and 0.5 M) after accumulating at 0.2 V for 150 s; (**B**) plot of the peak current versus H_2_SO_4_ concentration, error bars represent SD, *n* = 3; (**C**) the linear relationship between the peak potential and pH.

In order to maximize the electrochemical signal of 4-NP on the NC–PEI/GCE surface, various analytical parameters were optimized. To obtain the best supporting electrolyte for 4-NP detection, HCl, H_2_SO_4_, HNO_3_, H_3_PO_4_, HAc–NaAc (pH 4–6), HAc–NH_4_Ac (pH 4–6), phosphate buffer (pH 3–9) and Britton–Robinson (B–R) buffer (pH 3–9) (each 0.1 M) were selected as the supporting electrolytes for the test. The above supporting electrolytes were added to the solution containing 0.1 mM 4-NP, respectively, and the SDLSV curves were compared. The results show that the peak shape of 4-NP is the best, and the peak current is the largest in the H_2_SO_4_ solution. In addition, the acidity of the solution also has a significant effect on the electrochemical response of 4-NP. Since the determination is carried out in a H_2_SO_4_ solution, the pH value of the solution can be changed by changing the concentration of H_2_SO_4_. The electrochemical response of 4-NP at different concentrations of 0.02–0.5 M was tested (Figure 5A), and the results showed that in the concentration range of 0.2–0.5 M, the reduction current of 4-NP reached the maximum and was basically unchanged (Figure 5B). Therefore, we chose the concentration of 0.2 M in the following studies. In addition, Figure 5C shows that with pH increases from 0 to 1.5, the value of the 4-NP reduction peak potential (*E*_p_) decreases linearly, indicating that there was a proton transfer in the 4-NP reduction reaction, and the corresponding equation is as follows:

*E*_pa_ (V) = −0.05849 *pH* − 0.2676 (R^2^ = 0.9980) (2)

The slope of 58.49 mV is equal to the Nernst slope, indicating that the ratio of the number of electrons transferred to the number of reacting protons is equal to 1.

**Figure 6:**
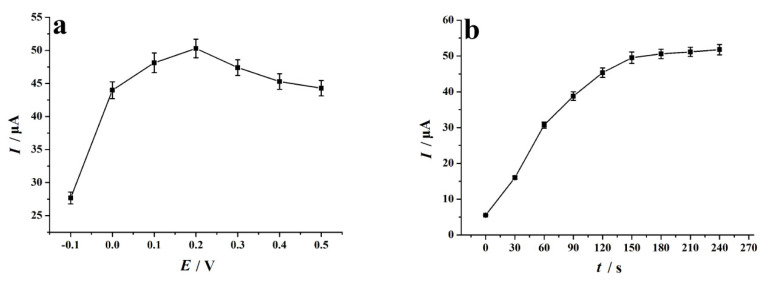
(**a**) Effect of accumulation potential on the peak current of 0.1 mM 4-NP after 150 s accumulation time; (**b**) effect of accumulation time on the peak current of 0.1 mM 4-NP at the accumulation potential of 0.2 V. Supporting electrolyte: 0.2 M H_2_SO_4_ solution, scan rate: 0.1 V s^−1^, error bars represent SD, *n* = 3.

We set different accumulation potential values in the range of −0.1 V to 0.5 V and studied the effect of accumulation potential on the electrochemical reduction behavior of 4-NP (accumulation time of 150 s). Figure 6a is the relationship diagram of the experimental data obtained: when the accumulation potential is 0.2 V, the reduction response peak of 4-NP on the NC–PEI/GCE sensor is the largest. Therefore, 0.2 V was selected as the best accumulation potential. In addition, the effect of accumulation time on the voltammetric behavior of 0.1 mM 4-NP on the NC–PEI/GCE surface was also studied (the accumulation potential was set at 0.2 V). Figure 6b shows a graph of the signal of the peak current relative to the accumulation time, indicating when the accumulation time is less than 150 s, the 4-NP reduction peak current is proportional to the accumulation time. After 150 s accumulation, the current was stable, and the equilibrium was reached. Therefore, the accumulation time was set to 150 s to obtain a better sensitivity for 4-NP detection.

### 3.5. Effect of Scan Rate 

**Figure 7:**
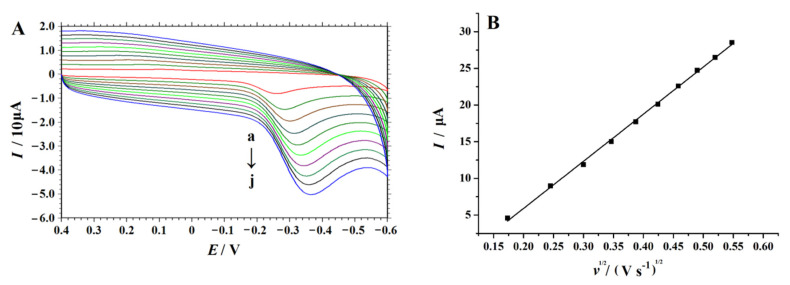
(**A**) Cyclic voltammograms obtained on the NC–PEI/GCE in 0.2 M H_2_SO_4_ solution containing 0.1 mM 4-NP under different scan rates (from a to j are 0.03, 0.06, 0.09, 0.12, 0.15, 0.18, 0.21, 0.24, 0.27 and 0.3 V s^−1^) after accumulating at 0.2 V for 30 s, (**B**) Linear relationship fitting between *I*_p_ and *v*^1/2^.

In order to study the kinetic process of an electrode reaction of 4-NP on the NC–PEI/GCE, the effect of the scan rate on the electrochemical behavior of 4-NP on the modified electrode was investigated by a CV technique. The cyclic voltammograms of 0.1 mM 4-NP in 0.2 M H_2_SO_4_ solution in the range of 30 to 300 mV s^−1^ were shown in Figure 7A. As shown in Figure 7B. when the square root of the sweep speed (ν^1/2^) value increases, the current intensity value increases linearly, and this equation was expressed as: *I*_p_ (μA) = 64.299 *v*^1/2^ − 6.9566 (R^2^ = 0.995). The result indicates that the diffusion-controlled electrode process of 4-NP occurs on the surface of NC–PEI/GCE in the studied range of the scan rate. 

### 3.6. Determination of 4-NP Using SDLSV

**Figure 8:**
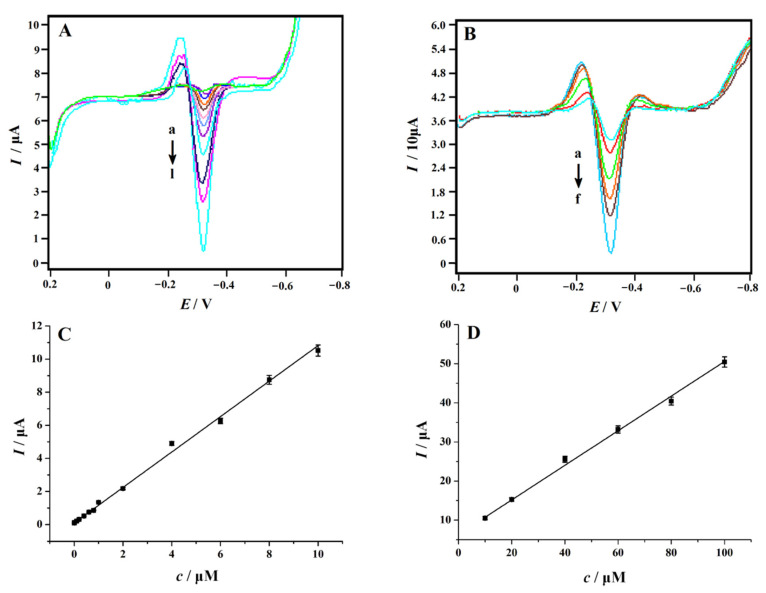
Second-order derivative linear sweep voltammograms of different concentrations of 4-NP recorded on the NC–PEI/GCE after accumulating at 0.2 V for 150 s. (**A**) Form a to l: 0.06, 0.08, 0.1, 0.2, 0.4, 0.6, 0.8, 1.0, 2.0, 4.0, 6.0, 8.0 and 10 μM 4-NP; (**B**) Form a to f: 10, 20, 40, 60, 80 and 100 μM 4-NP; (**C**) plots of the peak currents versus 4-NP concentrations in the range of 0.06–10 μM; and (**D**) plots of the peak currents versus 4-NP concentrations in the range of 10–100 μM, error bars represent SD, *n* = 3.

In order to obtain the linear range and detection limit of the NC–PEI/GCE in the measurement of 4-NP, an SDLSV technique was used to determine 4-NP at different concentrations in 0.2 M H_2_SO_4_ solution. The corresponding voltammograms were shown in Figure 8A,B. The peak current increased with the increase in concentration. Alternatively, as shown in Figure 8C,D, when the concentration of 4-NP increased in the range of 0.06–10 μM and 10–100 μM, the response of the sensor was linearly proportional to the concentration of 4-NP. The linear regression equations were: *I*_p_ (μA) = 1.0716 *c* (μM) + 0.1008 (R^2^ = 0.9985) and *I*_p_ (μA) = 0.4425 *c* (μM) + 6.3121 (R^2^ = 0.9963), respectively. The limit of detection (LOD) was estimated to be 0.01 μM (S/N = 3). Table 1 shows a comparison of the linear ranges and detection limits between some reported electrochemical sensors and NC–PEI/GCE in 4-NP detection. We can clearly recognize that the LOD obtained at the NC–PEI/GCE is lower than that of most reported modified electrodes, but not as good as that of RGO–MIP/GCE [22] and GR/ABPE [27]. However, the outstanding advantages of the NC–PEI/GCE are simple preparation, low price and fast response.

### 3.7. Investigation of the Selectivity

Some inorganic ions or organic substances were added to the 10 μM 4-NP solution as interfering substances to study the selectivity of NC–PEI/GCE. The tolerance level was considered to be the ratio of the interfering species concentration per 4-NP, resulting in a relative error of less than or equal to ±5.0%. The experimental results showed that 1000-fold concentrations of Na^+^, K^+^, Cu^2+^, Ca^2+^, Mg^2+^, Pb^2+^, Cd^2+^, Al^3+^, Cr^3+^ Mn^2+^, Cl^−^ and NO_3_^−^; a 100-fold concentration of phenol, hydroquinone and catechol; a 50-fold concentration of 2-aminophenol, 4-aminophenol, 2-chlorophenol and a two-fold concentration of 2-nitrophenol (2-NP) have no interference in the detection of 10 μM 4-NP, showing that the NC–PEI/GCE exhibits high selectivity in the detection of 4-NP.

### 3.8. Evaluation of the Repeatability, Reproducibility and Stability

The repeatability, reproducibility and stability of the NC–PEI/GCE were also investigated by SDLSV. Firstly, the voltammograms of 10 μM 4-NP in the 0.2 M H_2_SO_4_ solution were recorded for the same NC–PEI/GCE. The relative standard deviation (RSD) of the signals obtained for six repeated measurements was 2.7%, which indicated that the sensor had high repeatability in the determination of 4-NP. In addition, the reproducibility of the sensor was studied to evaluate the reliability of the electrode preparation method. Six different NC–PEI/GCEs were prepared by the same procedure, and the RSD was 3.4% for the detection of 10.0 μM 4-NP. NC–PEI/GCE also showed good stability without changes to the peak current after 200 cyclic voltammetric sweeps in the potential range between 0.2 and −0.6 V at the scan rate of 0.1 V s^−1^. The stability of the sensor was also evaluated by recording a signal of 10 μM 4-NP for the same NC–PEI/GCE every day. The results show that the modified electrode maintains about 93.2% of its initial current response after a week and maintains about 85.1% of its initial current response after a month. In conclusion, the NC–PEI/GCE has good analytical performance in repeatability, reproducibility and stability and can be used for the determination of 4-NP in real samples.

### 3.9. Analysis of the Real Sample

The suitability of NC–PEI/GCE for the 4-NP determination in water samples was studied. The water samples were determined without any pretreatment. For the analysis of the 4-NP content in real samples, an SDLSV technique and standard addition method were used. The results were shown in Table 2. No electrochemical response of 4-NP was found in any of the samples, which may be due to the absence of 4-NP in the water samples, or the concentration of 4-NP was lower than the detection limit of the method. A certain concentration of 4-NP was added, and the signal of SDLSV was recorded. The average value of four parallel measurements was adopted as the determination result. As shown in Table 2 through the spiked concentration of 4-NP in the water sample, the estimated recoveries obtained were between 96.0% and 104.7%. Therefore, this strategy can be successfully applied to the measurement of 4-NP in real samples.

## 4. Conclusions

In this study, a new C=N one-dimensional molecular chain can be established by reacting formamides with each other. Then, a novel N-doped carbon material (NC) with high nitrogen content was prepared by a step-by-step carbonization of the Schiff base. The electrochemical sensor base on NC–polyethyleneimine (PEI) composite shows excellent electrocatalytic activity to the 4-NP reduction on the electrode surface due to the excellent electronic, physical and chemical properties of the NC material, as well as the excellent dispersion, adsorption and binding properties of PEI. The diffusion-controlled electrode process of 4-NP occurs on the surface of NC–PEI/GCE, and the determination conditions were optimized. A wide linear range (0.06 μM to 100 μM) and a low detection limit (0.01 μM) were obtained for NC–PEI/GCE. Various environmental water samples were used to check the applicability of the NC–PEI/GCE. In addition, the sensors exhibit good repeatability, reproducibility and stability for 4-NP detection. Therefore, this simple strategy based on NC–PEI nanocomposite has potential application prospects in the design of other electrochemical sensors.

## Figures and Tables

**Table 1:** Performance comparison of the NC–PEI/GCE for 4-NP detection with other electrochemical sensors.

Technique	Sensor	Linear Range (μM)	Detection Limit (μM)	Reference
^a^ DPV	^e^ PNPI-PANI-PVSA/ITO	Not reported	1.0	[3]
DPV	^f^ CDSBA/CPE	0.2–1.6	0.01	[20]
^b^ LSV	^g^ GO/GCE	0.1–120	0.02	[21]
DPV	^h^ RGO–MIP/GCE	0.01–100	0.005	[22]
^c^ SWV	^i^ PAZ/Pt	30–90	8.23	[23]
DPV	^j^ PTTB/GCE	0.3–15	0.05	[24]
^d^ SDLSV	^k^ Gr–Chit/ABPE	0.1–80	0.08	[25]
SDLSV	^l^ S–CHIT/ABPE	0.08–2	0.03	[26]
SDLSV	^m^ GR/ABPE	0.02–100	0.008	[27]
SDLSV	NC–PEI/GCE	0.06–10; 10–100	0.01	This work

Note: ^a^ differential pulse voltammetry; ^b^ linear sweep voltammetry; ^c^ square wave voltammetry; ^d^ second-order linear scan voltammetry; ^e^ molecularly imprinted 4-nitrophenol sensor based on polyaniline–polyvinyl sulfonic acid composite film-modified indium tin oxide glass substrate; ^f^ β-cyclodextrin functionalized mesoporous silica-modified carbon paste electrode; ^g^ graphene oxide film-coated glassy carbon electrode; ^h^ graphene oxide and molecularly imprinted polymer-modified glassy carbon electrode; ^i^ polyazulene-modified platinum electrode; ^j^ thiophene-based microporous polymer film-modified glassy carbon electrode ^k^ graphene-chitosan composite-modified acetylene black paste electrode; ^l^ alicylaldehyde-modified chitosan-modified acetylene black paste electrode; ^m^ acetylene black paste and graphene hybrid electrode.

**Table 2:** Determination of 4-NP in water samples.

Samples ^a^	Added (μM)	Found ^b^ (μM)	RSD	Recovery (%)
River water	5.0	5.13	3.1	102.6
Lake water	10.0	10.38	2.8	103.8
Domestic sewage	40.0	38.86	3.7	97.2
Industrial waste water	20.0	20.94	4.6	104.7
Tap water	1.0	0.96	2.1	96.0

^a^ All water samples were collected from Hengyang, China. ^b^ Average value of four measurements.

## Data Availability

Not applicable.
